# Development of machine learning models with explainable AI for frailty risk prediction and their web-based application in community public health

**DOI:** 10.3389/fpubh.2025.1698062

**Published:** 2025-11-06

**Authors:** Seungmi Kim, Byung Kwan Choi, Jeong Su Cho, Up Huh, Myung-Jun Shin, Zoran Obradovic, Daniel J. Rubin, Jae Il Lee, Jong-Hwan Park

**Affiliations:** 1 Department of Convergence Medical Science, School of Medicine, Pusan National University, Yangsan, Republic of Korea; 2 Biomedical Research Institute, Pusan National University Hospital, Busan, Republic of Korea; 3 Department of Neurosurgery, Pusan National University School of Medicine, Yangsan, Republic of Korea; 4 Department of Neurosurgery, Pusan National University Hospital, Busan, Republic of Korea; 5 Department of Thoracic and Cardiovascular Surgery, Pusan National University School of Medicine, Yangsan, Republic of Korea; 6 Department of Thoracic and Cardiovascular Surgery, Pusan National University Hospital, Busan, Republic of Korea; 7 Department of Rehabilitation Medicine, Pusan National University School of Medicine, Yangsan, Republic of Korea; 8 Department of Rehabilitation Medicine, Pusan National University Hospital, Busan, Republic of Korea; 9 Center for Data Analytics and Biomedical Informatics, Temple University, Philadelphia, PA, United States; 10 Department of Computer and Information Sciences, Temple University, Philadelphia, PA, United States; 11 Lewis Katz School of Medicine, Temple University, Philadelphia, PA, United States; 12 Department of Convergence Medicine, Pusan National University School of Medicine, Yangsan, Republic of Korea; 13 Convergence Medical Institute of Technology, Pusan National University Hospital, Busan, Republic of Korea

**Keywords:** frailty, explainable AI, machine learning, SHAP, prediction model, digital health

## Abstract

**Background:**

Frailty is a public health concern linked to falls, disability, and mortality. Early screening and tailored interventions can mitigate adverse outcomes, but community settings require tools that are accurate and explainable. Korea is entering a super-aged phase, yet few approaches have used nationally representative survey data.

**Objective:**

This study aimed to identify key predictors of frailty risk using the K-FRAIL scale using explainable machine learning (ML), based on data from the 2023 National Survey of Older Koreans (NSOK). It also sought to develop and internally validate prediction models. To demonstrate the potential applicability of these models in community public health and clinical practice, a web-based application was implemented.

**Methods:**

Data from 10,078 older adults were analyzed, with frailty defined by the K-FRAIL scale (robust = 0, pre-frail = 1–2, and frail = 3–5). A total of 132 candidate variables were constructed through selection and derivation. Using CatBoost with out-of-fold (OOF) SHapley Additive exPlanations (SHAP, a game-theoretic approach to quantify feature contributions), 15 key predictors were identified and applied across 10 algorithms under nested cross-validation (CV). Model performance was evaluated using receiver operating characteristic–area under the curve (ROC-AUC), precision–recall area under the curve (PR-AUC), F1-score, balanced accuracy, and the Brier score. To assess feasibility, a single-page bilingual web application was developed, integrating the CatBoost inference pipeline for offline use.

**Results:**

SHAP analysis identified depression score, age, instrumental activities of daily living (IADL) count, sleep quality, and cognition as the leading predictors, followed by smartphone use, number of medications, province, driving status, hospital use, physical activity, osteoporosis, eating alone, digital adaptation difficulty, and sex, yielding 15 key predictors across the mental, functional, lifestyle, social, and digital domains. Using these predictors, boosting models outperformed other algorithms, with CatBoost achieving the best performance (ROC-AUC = 0.813 ± 0.014; PR-AUC = 0.748 ± 0.019).

**Conclusion:**

An explainable machine learning model with strong discrimination performance and adequate calibration was developed, accompanied by a lightweight web application for potential use in community and clinical settings. However, external validation, recalibration, and subgroup fairness assessments are needed to ensure generalizability and clinical adoption.

## 1 Introduction

Frailty is a clinical and public health condition characterized by reduced physiological reserve and diminished resistance to stress, leading to increased risks of falls, hospitalization, disability, and mortality (1–4). With the acceleration of global population aging, the burden of frailty is steadily rising, highlighting the need for early screening and preventive interventions at both national and regional levels (5, 6).

However, large-scale surveys and clinical data are inherently complex, often exhibiting non-linearity, interactions, missingness, and heterogeneity. Such characteristics limit the predictive accuracy and interpretability of traditional linear models (7, 8). Machine learning methods can capture these complex structures, yet their limited explainability has constrained their acceptance in public health and clinical practice.

Explainable artificial intelligence has emerged as a promising approach to address this gap. Among these methods, SHapley Additive exPlanations is a game-theoretic technique that quantifies the contribution of each variable to model predictions and enables the intuitive interpretation of results (9–11). By providing transparency at global and individual levels, it enhances the trustworthiness of predictive models.

In imbalanced binary classification problems, receiver operating characteristic metrics alone are insufficient. The precision–recall curves and the precision–recall area under the curve (PR-AUC), which summarize the balance between precision and recall across thresholds, provide complementary information, and the Youden index can guide threshold selection (12–14). Moreover, the Brier score, the mean squared difference between predicted probabilities and observed outcomes with lower values indicating better calibration, should be reported to support the clinical interpretability of probability predictions (15, 16).

Reducing bias in internal validation also requires an appropriate cross-validation design. Nested cross-validation, a two-layer procedure in which the inner loop performs model selection while the outer loop yields an unbiased performance estimate, is recommended to separate these processes and ensure robust evaluation (17, 18).

In this study, data from the 2023 Korean National Survey of Older Koreans were used to develop a binary classifier that distinguishes robust (0) from pre-frail (1–2)/frail (3–5) individuals. A globally fixed set of the top 15 features was selected using out-of-fold SHapley Additive exPlanations and applied consistently across 10 algorithms. Performance was evaluated within a nested cross-validation framework. The results on discrimination, calibration, and explainability are reported, and a web-based application is further proposed to demonstrate practical implementation (9, 10, 15, 17–28).

## 2 Methods

### 2.1 Data source/study population

A cross-sectional study was conducted using the 2023 NSOK (29, 30). The Ministry of Health and Welfare and the Korea Institute for Health and Social Affairs led the survey (29). The NSOK targets all Koreans aged ≥65 years living in general households, excluding island enumeration areas (EAs), collective facilities (e.g., dormitories and nursing homes), tourist hotels, foreigner EAs, and non-household residents (e.g., overseas residents, active-duty military, and incarcerated people). Sampling used explicit three-stage stratification—17 provinces, urbanicity (dong vs. eup/myeon), and EA type (apartment vs. general)—followed by probability-proportional-to-size (PPS) selection of EAs, systematic sampling of households within selected EAs, and full enumeration of all eligible residents ≥65 years within sampled households (30–32). Trained interviewers collected data via tablet-assisted personal interviews during home visits.

The final sample included 10,078 participants, comprising 6,324 robust (62.7%), 3,313 pre-frail (32.9%), and 441 frail (4.4%) individuals. Design, non-response, within-household, and post-stratification adjustments (region × sex × age) were used to construct final sampling weights; however, weights were not applied for model development, and descriptive statistics reflected the unweighted sample distribution (33, 34). Given the relatively small prevalence of the frail group, population estimates for this subgroup should be interpreted cautiously, and our primary inference concerns predictive performance within the sample.

The dataset consisted of de-identified, nationally approved statistics. The study was exempted from institutional review board review (exemption ID: PNU IRB/2025_161_HR).

### 2.2 Variables and feature engineering

From 661 raw survey variables, 132 predictors aligned with the study objectives were constructed. Domains included sociodemographics; physical health (32 physician-diagnosed chronic conditions, anthropometrics, number of medications, hospital use, falls, health checkups, and unmet medical needs); health behaviors (smoking, alcohol, physical activity, diet, and sleep); mental health (15-item depression scale and suicidal ideation); cognition (total score); activities of daily living (ADL/IADL as binary indicators and counts); medical and care use; social activity and life satisfaction; and digital capacity (35–39). Variables directly or indirectly defining K-FRAIL items or the frailty target were excluded to prevent information leakage (40).

“Not applicable/no response” and unrealistic special codes (e.g., 9,998, and 99,999) were recoded as missing according to the official codebook, and cognitive scores were converted to the numeric type. ADL/IADL limitations were binarized, and limitation counts were computed. Body mass index (BMI) was derived from height and weight. After these derivations, the final predictor set comprised 132 variables (118 original and derived variables such as ADL_Count, IADL_Count, and BMI), all of which were used for model development (Supplementary Table S3).

### 2.3 Preprocessing

All preprocessing for model training was implemented within a single pipeline to avoid information leakage during cross-validation. Missing values were imputed independently within each training fold: continuous variables with the median and categorical variables with the mode. Categorical predictors were defined according to an *a priori* codebook and treated as either ordinal or nominal. Ordinal variables were encoded with preserved order using *OrdinalEncoder*, whereas nominal variables were encoded with *OneHotEncoder* after imputation. Continuous variables (e.g., age, BMI, income, cognitive score, and depression score) were standardized using *StandardScaler*. Ordinal variables were encoded but not scaled because their rank information was preserved directly. Scaling was thus restricted to continuous inputs and applied only for algorithms sensitive to feature magnitude (e.g., support vector machine (SVM), k-nearest neighbors (KNN), multilayer perceptron (MLP), and logistic regression). Tree-based methods (random forest, gradient boosting, XGBoost, LightGBM, and CatBoost) were trained without scaling. Class imbalance was addressed within each training fold by applying the Synthetic Minority Over-sampling Technique for Nominal and Continuous (SMOTENC) features using the categorical feature indices.

### 2.4 Outcome definition

The outcome was based on the K-FRAIL scale, which sums five items—fatigue, difficulty climbing stairs, difficulty walking 300 m, number of chronic diseases, and weight loss—each coded 0/1 to yield a score of 0–5 (41, 42). The primary analysis was binary classification (robust = 0 vs. pre-frail/frail = 1–5). For descriptive comparisons, three groups were also defined: robust (0), pre-frail (1–2), and frail (3–5).

In the original FRAIL scale, the weight-loss item is defined as a loss of 5% or more of body weight over the prior 12 months, and the K-FRAIL adopted this criterion. In the 2023 survey, however, no variable measuring a 12-month 5% weight loss was available. Instead, a variable capturing unintentional weight change of 5 kg or more during the prior 6 months was available. Accordingly, this 6-month weight-change variable (loss or gain) was used as a proxy for the original item. This operational difference from the standard definition should be taken into account when interpreting the results.

### 2.5 Explainability analysis

**Figure 1:**
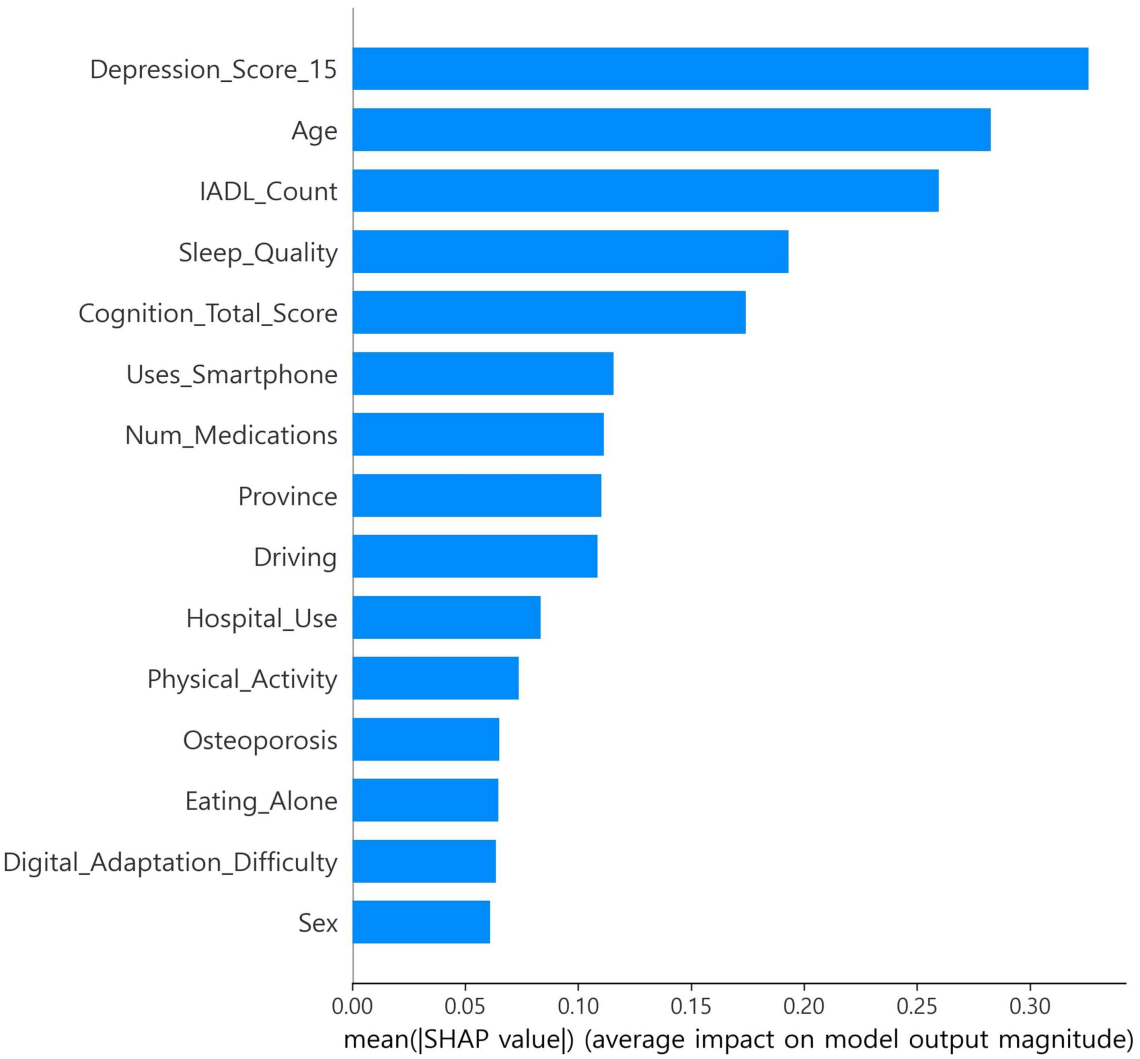
SHAP feature importance.

**Figure 2:**
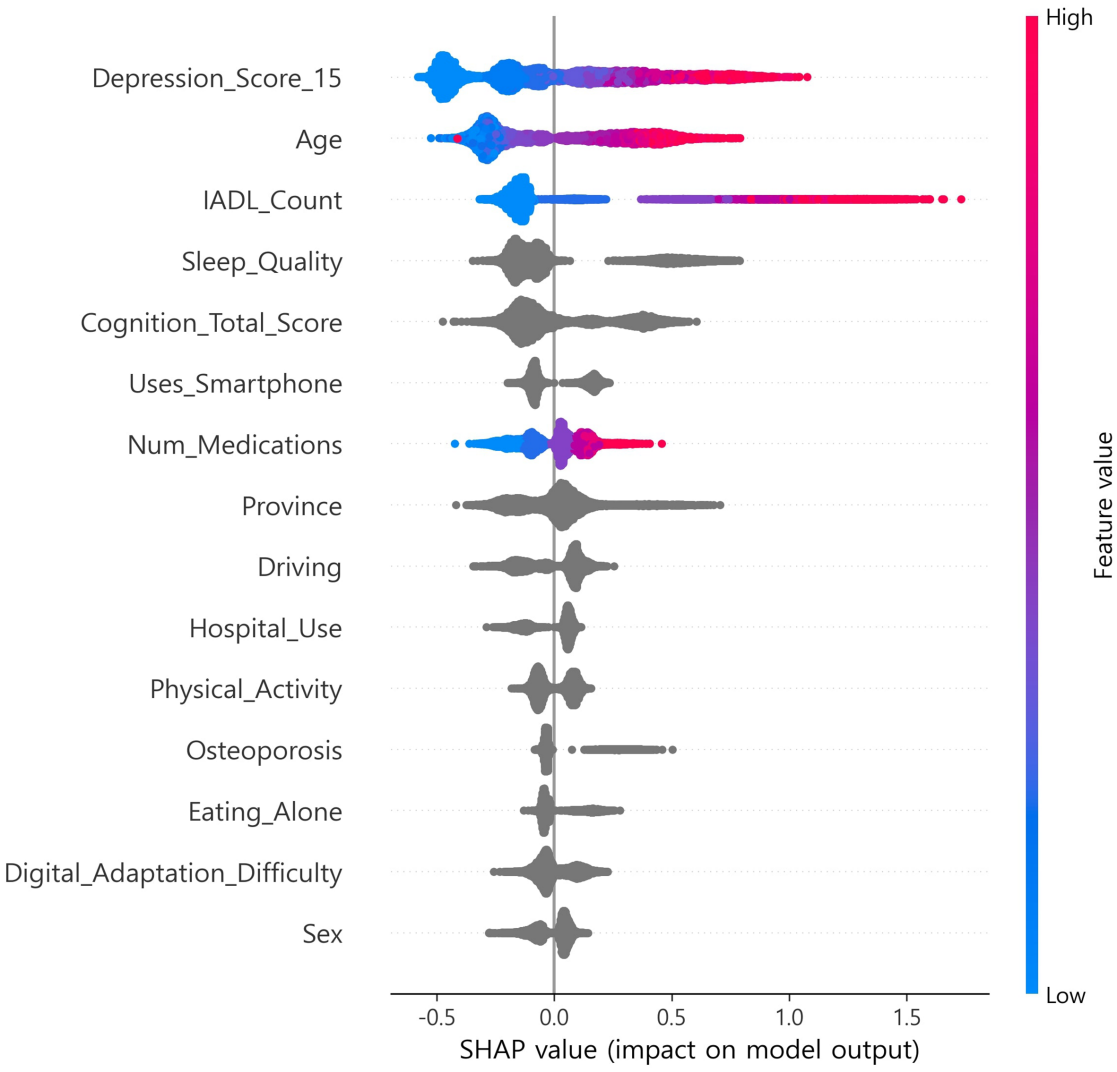
SHAP beeswarm plot.

SHapley Additive exPlanations (SHAP), which estimates feature contributions based on cooperative game theory, was applied, and out-of-fold (OOF) SHAP was performed using a single CatBoost classifier to support feature selection and interpretability (9, 10, 23). Stratified k-fold (default 5-fold) cross-validation was used, and SHAP values were computed on each fold’s validation data and then aggregated at the out-of-fold (OOF) level (9, 10, 17, 18). Features were ranked by mean absolute SHAP. *In exploration, the top 15 features were selected once from the full-sample OOF-SHAP.* This set was then fixed globally for all algorithms and across all nested cross-validation steps (17, 18). Global summary bar plots and local beeswarm plots were generated. The OOF-SHAP matrix and the final top 15 list were saved for reproducibility (Figures 1, 2) (9, 10).

### 2.6 Machine learning models and training

A binary classifier was built to distinguish robust (0) from pre-frail/frail (1). We implemented ten supervised learning algorithms, representing major categories of machine learning: linear (logistic regression), kernel-based (support vector machine with radial basis function (RBF) kernel), instance-based (k-nearest neighbors), neural network (multilayer perceptron), and tree-based ensemble methods (random forest, gradient boosting, histogram-based gradient boosting, XGBoost, LightGBM, and CatBoost) (21–28).

To ensure unbiased performance estimation, nested cross-validation with an outer stratified 10-fold split and an inner stratified 5-fold split was used (17, 18). Model hyperparameters were optimized in the inner loop using the ROC-AUC as the selection criterion. For each outer fold, the configuration yielding the highest ROC-AUC was chosen. For some algorithms (e.g., logistic regression, random forest, and boosting methods), the class_weight parameter (none vs. balanced) was additionally searched.

For each outer fold, the optimal classification threshold was selected based on the inner cross-validation results by maximizing Youden’s J index (sensitivity + specificity − 1). The fold-specific threshold was then applied to the corresponding outer test fold, and final performance metrics were aggregated across folds (13, 14). To ensure reproducibility, random_state was set to 42.

Primary metrics were accuracy, sensitivity, specificity, precision, F1 score, balanced accuracy, PR-AUC, ROC-AUC, and the Brier score. Outer-fold results were summarized as mean ± SD. A 95% CI for ROC-AUC was computed from 2,000 bootstrap samples using all OOF probabilities (43). Calibration was assessed using decile-based calibration plots (15).

### 2.7 Web application implementation

To assess field feasibility, a single-page web application was built. The application integrates the trained CatBoost inference pipeline with a bilingual (Korean/English) questionnaire schema. It standardizes responses according to the codebook, with categorical variables integer-coded and continuous variables converted to numeric type, aligns inputs to the model’s feature set, and treats missing entries as missing (29). The CatBoost classifier outputs the predicted probability for the positive (frailty) class and assigns a final label by comparing the probability with a predefined or user-adjustable threshold (13, 14). All inference is executed locally without server connection, and predictions together with metadata (timestamp, probability, threshold, and label) are stored in local files (CSV/XLSX).

## 3 Results

### 3.1 Baseline characteristics of participants

Data from 10,078 participants were included (robust: 62.7%, pre-frail: 32.9%, and frail: 4.4%; Table 1). The majority of continuous variables differed significantly across groups (age, number of medications, depression score, cognitive score, and household income; all *p* < 0.001), while the BMI showed a smaller but significant difference (*p* = 0.031). The majority of categorical variables also showed significant differences (sex, age group, self-rated health, ADL/IADL dependency, physical activity, hospital use, health checkups, fall experience, smartphone use, and digital adaptation difficulty; mostly a *p*-value of < 0.001). For interpretability, categories were reordered so that higher numeric values corresponded to worse clinical meaning for items such as self-rated health, sleep quality, and digital adaptation difficulty. Because the frail group represented only 4.4% of the sample, some category estimates may remain unstable, and descriptive interpretations are presented conservatively. See Table 1 and Supplementary Table S1 for details.

**Table 1:** Baseline characteristics of participants by frailty group.

Variable	Total (*N* = 10,078, 100%)	Robust (*n* = 6,324, 62.7%)	Pre-frail (*n* = 3,313, 32.9%)	Frail (*n* = 441, 4.4%)	*p*-value
Continuous variables (mean ± SD)					
Age	74.13 ± 6.81	72.35 ± 6.01	76.89 ± 7.04	78.83 ± 6.66	<0.001
BMI	23.63 ± 2.63	23.68 ± 2.41	23.54 ± 2.93	23.53 ± 3.29	0.031
Number of medications	2.05 ± 1.62	1.72 ± 1.42	2.48 ± 1.72	3.48 ± 1.97	<0.001
Depression score	3.06 ± 3.23	2.23 ± 2.59	4.05 ± 3.44	7.42 ± 4.21	<0.001
Cognitive score	24.50 ± 4.77	25.57 ± 4.20	22.87 ± 5.01	21.07 ± 5.55	<0.001
Household income	3251.7 ± 3412.9	3617.4 ± 3509.4	2702.9 ± 3238.1	2131.5 ± 2327.3	<0.001
Categorical variables (*n*, %)					
Sex					<0.001
Male	3,872 (38.4)	2,710 (42.9)	1,047 (31.6)	115 (26.1)	
Female	6,206 (61.6)	3,614 (57.1)	2,266 (68.4)	326 (73.9)	
Age group					<0.001
65–69	3,249 (32.2)	2,580 (40.8)	623 (18.8)	46 (10.4)	
70–74	2,482 (24.6)	1739 (27.5)	666 (20.1)	77 (17.5)	
75–79	1950 (19.3)	1,091 (17.3)	763 (23.0)	96 (21.8)	
80–84	1,544 (15.3)	655 (10.4)	758 (22.9)	131 (29.7)	
85–89	668 (6.6)	217 (3.4)	383 (11.6)	68 (15.4)	
≥90	185 (1.8)	42 (0.7)	120 (3.6)	23 (5.2)	
Self-rated health					<0.001
Excellent	240 (2.4)	209 (3.3)	31 (0.9)	0 (0.0)	
Good	3,873 (38.4)	3,052 (48.3)	790 (23.8)	31 (7.0)	
Fair	3,445 (34.2)	2,277 (36.0)	1,099 (33.2)	69 (15.6)	
Poor	2,107 (20.9)	738 (11.7)	1,139 (34.4)	230 (52.2)	
Very poor	286 (2.8)	30 (0.5)	158 (4.8)	98 (22.2)	
Missing	127 (1.3)	18 (0.3)	96 (2.9)	13 (2.9)	
ADL dependency					<0.001
Yes	820 (8.1)	151 (2.4)	502 (15.2)	167 (37.9)	
No	9,258 (91.9)	6,173 (97.6)	2,811 (84.8)	274 (62.1)	
IADL dependency					<0.001
Yes	1762 (17.5)	503 (8.0)	1,001 (30.2)	258 (58.5)	
No	8,316 (82.5)	5,821 (92.0)	2,312 (69.8)	183 (41.5)	
Physical activity					<0.001
Yes	5,376 (53.3)	3,717 (58.8)	1,512 (45.6)	147 (33.3)	
No	4,702 (46.7)	2,607 (41.2)	1801 (54.4)	294 (66.7)	
Hospital use (past year)					<0.001
Yes	6,982 (69.3)	4,042 (63.9)	2,563 (77.4)	377 (85.5)	
No	3,096 (30.7)	2,282 (36.1)	750 (22.6)	64 (14.5)	
Health checkup					<0.001
Yes	7,920 (78.6)	5,203 (82.3)	2,389 (72.1)	328 (74.4)	
No	2,158 (21.4)	1,121 (17.7)	924 (27.9)	113 (25.6)	
Fall experience					<0.001
Yes	639 (6.3)	209 (3.3)	338 (10.2)	92 (20.9)	
No	9,439 (93.7)	6,115 (96.7)	2,975 (89.8)	349 (79.1)	
Owns smartphone					<0.001
Yes	7,499 (74.4)	5,264 (83.2)	2021 (61.0)	214 (48.5)	
No	2,579 (25.6)	1,060 (16.8)	1,292 (39.0)	227 (51.5)	
Uses smartphone					<0.001
Yes	6,432 (63.8)	4,723 (74.7)	1,561 (47.1)	148 (33.6)	
No	3,646 (36.2)	1,601 (25.3)	1752 (52.9)	293 (66.4)	
Digital adaptation difficulty					<0.001
Very easy	61 (0.6)	47 (0.7)	14 (0.4)	0 (0.0)	
Easy	640 (6.4)	498 (7.9)	133 (4.0)	9 (2.0)	
Moderate	2,319 (23.0)	1771 (28.0)	512 (15.5)	36 (8.2)	
Difficult	4,075 (40.4)	2,720 (43.0)	1,245 (37.6)	110 (24.9)	
Very difficult	2,856 (28.3)	1,270 (20.1)	1,313 (39.6)	273 (61.9)	
Missing	127 (1.3)	18 (0.3)	96 (2.9)	13 (2.9)	

Continuous variables were compared using a one-way ANOVA or the Kruskal–Wallis test depending on normality, and categorical variables were compared using the chi-squared (χ^2^) test.

### 3.2 Explainability analysis

The CatBoost OOF-SHAP top 15 ranked the 15-item depression score as the most important predictor by mean absolute SHAP, followed by age, IADL count, sleep quality, and total cognition score (Figure 1). Other contributing features included smartphone use, number of medications, province, driving, hospital use, physical activity, osteoporosis, eating alone, digital adaptation difficulty, and sex. The SHAP beeswarm plot (Figure 2) showed that higher depression scores, older age, more IADL limitations, poorer sleep quality, and lower cognition were strongly associated with increased frailty risk. Additional risk was linked to not using a smartphone, polypharmacy, eating alone, greater difficulty with digital adaptation, and being female. In contrast, higher cognition, physical activity, and absence of IADL limitations were associated with lower risk.

### 3.3 Model performance

**Figure 3:**
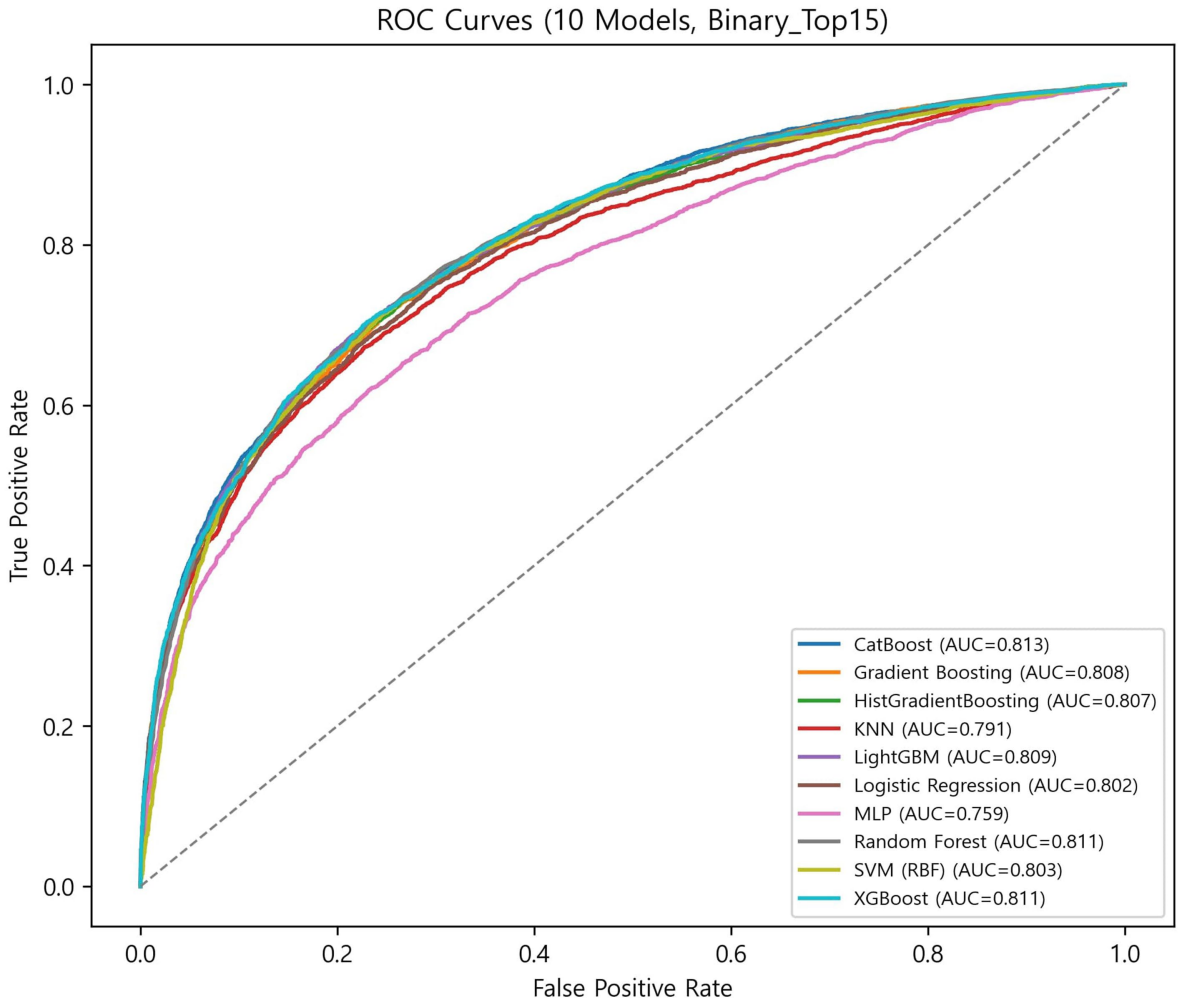
ROC curves of 10 models.

**Figure 4:**
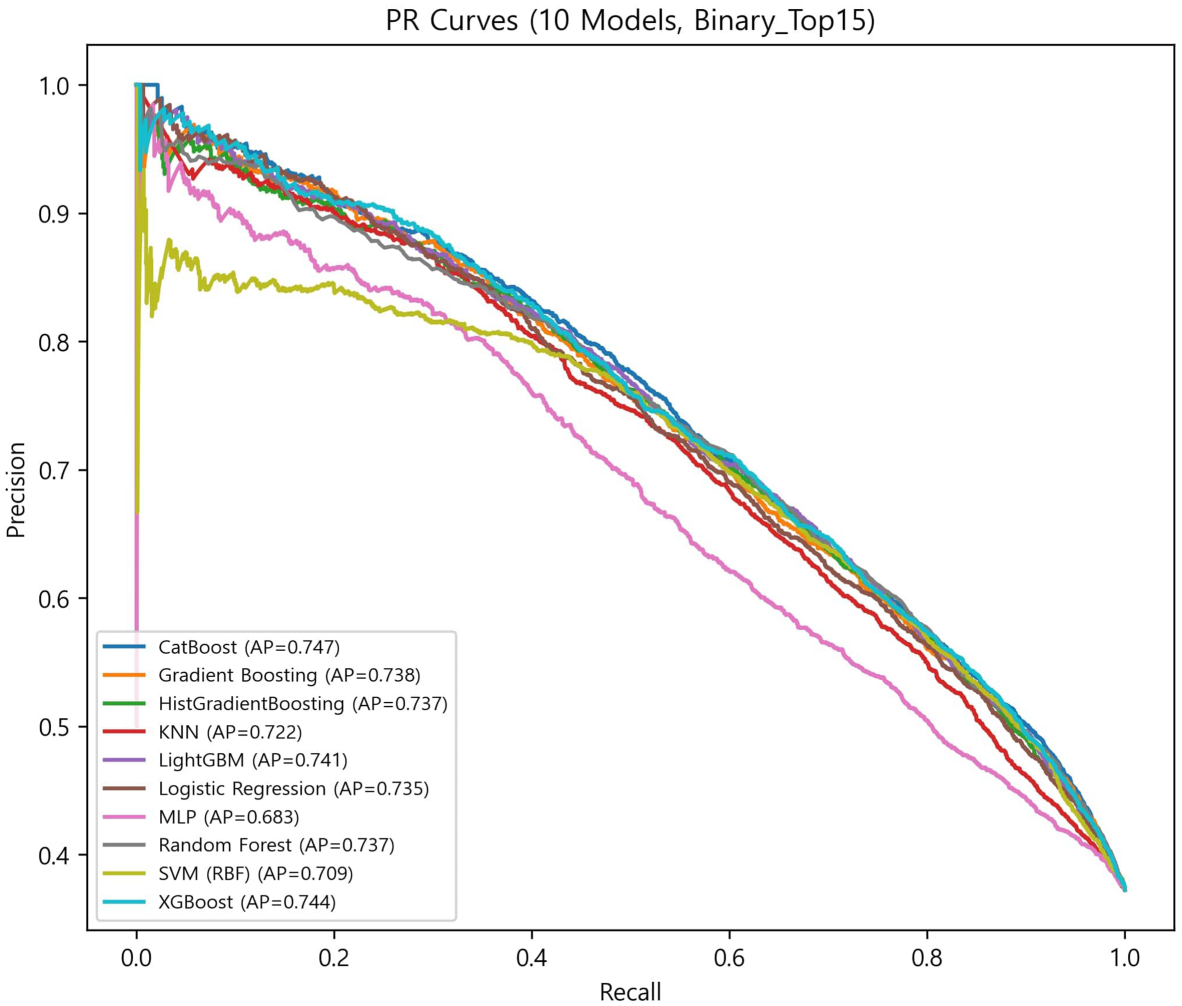
PR curves of 10 models.

The globally fixed top 15 features derived from the CatBoost OOF-SHAP analysis were consistently applied across all algorithms and outer folds. Nested cross-validation (outer 10-fold and inner 5-fold) demonstrated that tree-based ensemble models achieved the best overall performance (Figures 3, 4, Table 2).

**Table 2:** Performance of 10 machine learning models for binary frailty classification.

Model	Balanced accuracy	Sensitivity	Specificity	Accuracy	Precision	F1-score	Brier score	ROC-AUC	PR-AUC
Logistic regression	0.726 ± 0.015	0.691 ± 0.035	0.761 ± 0.027	0.735 ± 0.015	0.632 ± 0.022	0.660 ± 0.019	0.178 ± 0.006	0.803 ± 0.014	0.736 ± 0.017
SVM (RBF)	0.733 ± 0.014	0.705 ± 0.024	0.760 ± 0.027	0.740 ± 0.015	0.636 ± 0.022	0.669 ± 0.015	0.177 ± 0.006	0.803 ± 0.012	0.711 ± 0.018
KNN	0.722 ± 0.015	0.678 ± 0.027	0.766 ± 0.026	0.733 ± 0.016	0.633 ± 0.024	0.654 ± 0.018	0.181 ± 0.007	0.791 ± 0.016	0.724 ± 0.020
MLP	0.692 ± 0.016	0.630 ± 0.054	0.753 ± 0.041	0.708 ± 0.015	0.605 ± 0.025	0.615 ± 0.024	0.212 ± 0.009	0.760 ± 0.013	0.686 ± 0.018
Random forest	0.733 ± 0.012	0.699 ± 0.029	0.768 ± 0.028	0.742 ± 0.013	0.642 ± 0.023	0.669 ± 0.014	0.168 ± 0.004	0.811 ± 0.010	0.738 ± 0.015
Gradient boosting	0.730 ± 0.017	0.697 ± 0.037	0.764 ± 0.036	0.739 ± 0.018	0.638 ± 0.028	0.665 ± 0.020	0.168 ± 0.006	0.808 ± 0.013	0.740 ± 0.021
HistGradientBoosting	0.733 ± 0.017	0.702 ± 0.038	0.765 ± 0.030	0.741 ± 0.017	0.640 ± 0.026	0.669 ± 0.021	0.168 ± 0.006	0.807 ± 0.012	0.740 ± 0.018
XGBoost	0.736 ± 0.019	0.704 ± 0.033	0.768 ± 0.030	0.744 ± 0.020	0.644 ± 0.029	0.672 ± 0.023	0.166 ± 0.006	0.811 ± 0.013	0.745 ± 0.021
LightGBM	0.733 ± 0.017	0.707 ± 0.040	0.759 ± 0.027	0.740 ± 0.015	0.636 ± 0.023	0.669 ± 0.021	0.167 ± 0.006	0.809 ± 0.014	0.743 ± 0.019
CatBoost	0.732 ± 0.016	0.692 ± 0.035	0.771 ± 0.032	0.742 ± 0.017	0.644 ± 0.026	0.666 ± 0.019	0.165 ± 0.006	0.813 ± 0.014	0.748 ± 0.019

Among ensemble models, CatBoost achieved the highest discrimination (ROC-AUC = 0.813 ± 0.014; PR-AUC = 0.748 ± 0.019) and the best calibration (specificity = 0.771 ± 0.032; Brier = 0.165 ± 0.006). XGBoost showed the highest balanced overall performance (balanced accuracy = 0.736 ± 0.019; accuracy = 0.744 ± 0.020; and F1-score = 0.672 ± 0.023). LightGBM achieved the highest sensitivity (0.707 ± 0.040) with balanced overall metrics (accuracy = 0.740 ± 0.015 and specificity = 0.759 ± 0.027). Random forest demonstrated stable and low-variance performance (balanced accuracy = 0.733 ± 0.012; accuracy = 0.742 ± 0.013; and Brier = 0.168 ± 0.004). Gradient boosting and HistGradientBoosting yielded comparable results, with ROC-AUC values of 0.807–0.808 and PR-AUC approximately 0.740.

In contrast, simpler models, such as the logistic regression analysis, SVM, and KNN, achieved moderate performance (ROC-AUC = 0.791–0.803 and PR-AUC = 0.711–0.736), while MLP showed the lowest overall performance (balanced accuracy = 0.692 ± 0.016; ROC-AUC = 0.760 ± 0.013; and Brier = 0.212 ± 0.009).

Overall, ensemble models demonstrated similar discrimination, with ROC-AUC ranging from 0.807 to 0.813 and PR-AUC ranging from 0.738 to 0.748. Brier scores ranged from 0.165 to 0.181, indicating generally good probability calibration. Calibration curves (Supplementary Figure S1) were closely aligned with the diagonal across models, although the MLP showed larger deviations at low and high predicted probabilities.

The CatBoost OOF confusion matrix (Supplementary Figure S2) showed TN = 5,385, FP = 939, FN = 1,490, and TP = 2,264, corresponding to sensitivity = 0.603, specificity = 0.852, and balanced accuracy = 0.728. These OOF-level metrics were comparable to the nested CV averages in Table 2, confirming consistency between OOF and cross-validation results.

### 3.4 Web application implementation

**Figure 5:**
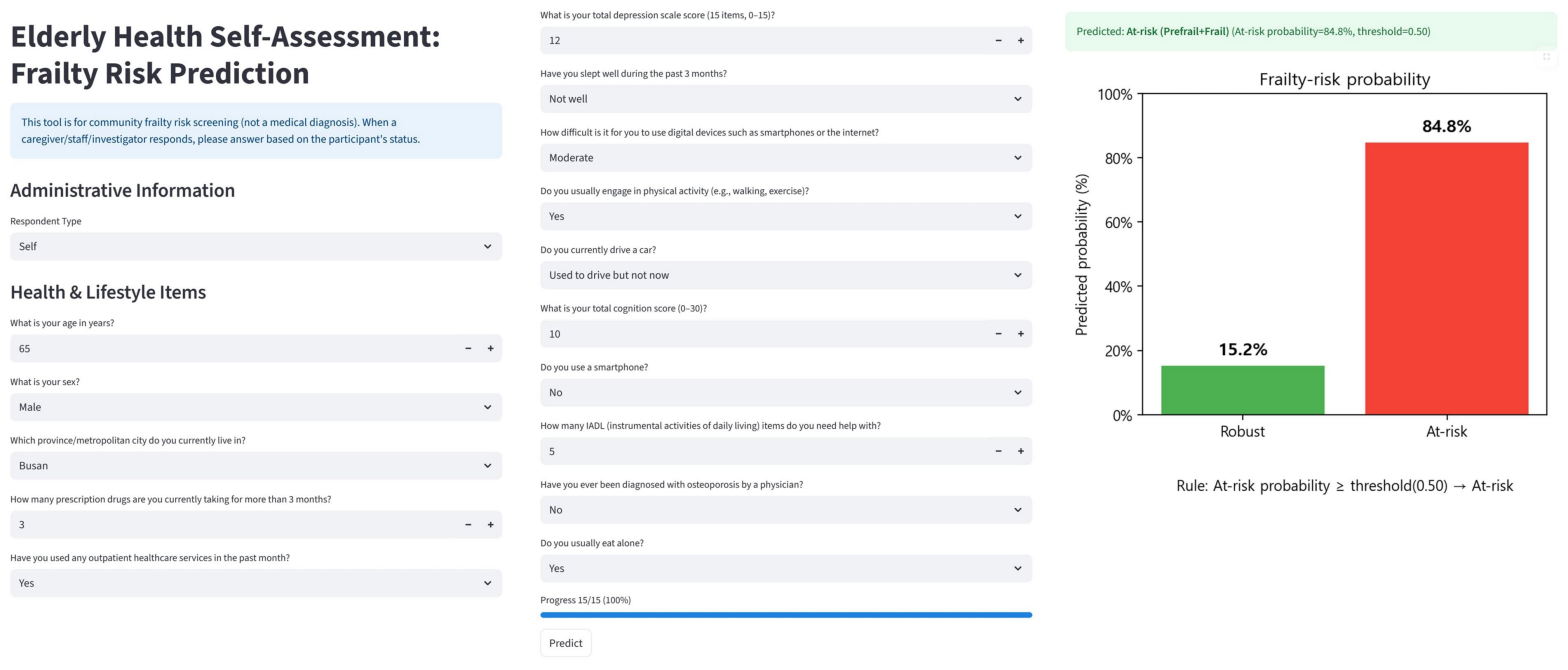
Web-based frailty prediction application interface.

The CatBoost-based trained pipeline and globally fixed top 15 features were implemented in a single-page bilingual (Korean/English) web application (Figure 5). The interface consists of three components: (i) administrative information, (ii) health and lifestyle questionnaire items corresponding to the top 15 predictors, and (iii) probability output with visualization.

In the design, the administrative information fields (e.g., respondent type, age, sex, and residential area) are used to record participant characteristics. Since some of these overlap with the top 15 predictors, they can be streamlined or replaced so that the information is directly reflected in the model input, ensuring consistency between the questionnaire and prediction pipeline. User inputs are standardized according to the official codebook (e.g., integer coding for categorical variables and numeric casting for continuous variables) and aligned with the model’s feature set. Missing values are handled as defined during training.

After submission, the classifier outputs the predicted frailty probability for robust and at-risk categories and assigns a label using a default threshold of 0.50, which can be adjusted by the user. The results are visualized as bar charts showing class probabilities and final risk classification. The application also stores inputs, predictions, and metadata (time, threshold, and label) using browser localStorage and client-side file download (CSV/XLSX), enabling cumulative tracking. This design supports rapid screening, intuitive communication of risk, and community-level monitoring, bridging explainable machine learning models with practical public health applications.

## 4 Discussion

### 4.1 Model performance and calibration

Ensemble learning approaches consistently demonstrated superior discrimination compared with traditional classifiers, consistent with prior studies showing that tree-based boosting algorithms effectively learn non-linear relationships in complex data (21–24). The narrow range of ROC-AUC values (0.807–0.813) across CatBoost, XGBoost, and LightGBM indicates stable performance among ensemble methods, suggesting that proper variable representation and calibration are more critical than the specific boosting framework itself.

In contrast, linear and kernel-based classifiers such as logistic regression and SVM achieved moderate discrimination (ROC-AUC = 0.791–0.803), indicating limited ability to capture the complexity and heterogeneity of frailty-related health determinants (21–24).

Low Brier scores (0.165–0.181) and calibration curves closely aligned with the diagonal (Supplementary Figure S1) indicate reliable probability estimation (15, 16). CatBoost, in particular, achieved the highest discrimination (ROC-AUC = 0.813 ± 0.014) and the best calibration performance (Brier = 0.165 ± 0.006), demonstrating that explainable ensemble models can simultaneously achieve high predictive accuracy and interpretable probability estimates when applied to large-scale survey and community health datasets (9, 10, 15, 16, 21–24).

### 4.2 Explainability and interpretation

The SHAP-based interpretation demonstrates that frailty is a multidimensional construct resulting from interactions among mental, functional, social, and digital domains. Core predictors such as depression, cognitive function, sleep quality, and IADL limitations highlight how psychological and functional decline jointly contribute to physical vulnerability (9, 10, 35–37, 44, 45). This aligns with previous evidence that frailty is not merely a physiological condition but a complex syndrome shaped by multiple interdependent factors.

In addition, social and digital factors—including smartphone use, eating alone, and difficulty adapting to technology—underscore the evolving relevance of social connectivity and digital capacity in aging populations (38, 39, 46–50). These variables illustrate how modern forms of exclusion, both social and digital, can amplify vulnerability among older adults.

Together, these findings expand upon the multidimensional framework of frailty proposed in earlier studies (1, 2, 35–37, 44–47), emphasizing that frailty should be understood as a cumulative state of vulnerability across psychological, functional, social, and digital dimensions. From a public health perspective, this multidomain understanding highlights the importance of integrating mental, physical, and social support systems within community-based aging policies.

### 4.3 Clinical and public health implications

This study demonstrated that the majority of the major predictors of frailty risk identified by the model are actionable factors. Depression, poor sleep quality, and polypharmacy emerged as leading predictors, suggesting that routine mental health screening, sleep hygiene education, and systematic medication review should be incorporated into primary care and community-based aging programs (35, 44, 45). In addition, promotion of physical activity and cognitive training may represent practical strategies to mitigate the progression of frailty (36, 46, 47).

Beyond the individual level, social and behavioral factors such as eating alone (48, 49), digital adaptation difficulty, and medical conditions such as osteoporosis (50) were also associated with frailty risk, reflecting structural and clinical challenges faced by aging societies. These findings indicate that frailty prediction can be meaningfully linked to broader public health approaches, including digital literacy programs for older adults, social participation initiatives, and targeted support for vulnerable groups (37, 51). Difficulty in digital adaptation emerged as an indicator closely linked to health equity, as it may exacerbate disparities in healthcare access and information utilization (37, 51). Ensuring digital equity should therefore be regarded as an important public health priority in the management of aging populations (5, 6).

Finally, the lightweight web application developed in this study enables frontline health providers to rapidly identify high-risk individuals and intuitively communicate results, thereby bridging technical outputs with practical counseling and community health planning. Such an approach aligns with the WHO and United Nations Decade of Healthy Ageing 2021–2030 initiatives (52, 53) and illustrates the potential for international scalability of explainable frailty prediction models.

### 4.4 Strengths

Using national-scale survey data, SHAP-based analysis was applied to select candidates, and a globally fixed feature set was constructed (9, 10). With this set, ten algorithms were compared using nested CV to ensure fairness, improve reproducibility, and assess both discrimination and calibration (Table 2) (17, 18, 54). Global and local interpretability were presented with SHAP (Figures 1, 2) (9, 10). A lightweight model was also embedded in a web environment to demonstrate field applicability (Figure 5).

### 4.5 Limitations

First, the cross-sectional design precludes causal inference; associations are predictive correlations (1,2,6). Second, self-reported mental health, behaviors, and digital capacity may suffer from measurement error and social desirability bias (32). Third, the frail group was only 4.4%, which can inflate uncertainty; residual bias may remain despite SMOTENC (19, 20). Fourth, although the NSOK is nationally representative by design, sampling weights were not applied in model estimation, and descriptive statistics were unweighted. Consequently, the results should not be interpreted as population-level estimates; they reflect predictive performance in the observed sample. Fifth, selecting and fixing features once with full-sample OOF-SHAP improves reproducibility but risks underestimating fold- or algorithm-specific features (17, 18). Sixth, subgroup performance and fairness (sex, age strata, socioeconomic status, and digital divide) were not comprehensively assessed (51). Seventh, the FRAIL/K-FRAIL weight-loss item is defined as a reduction of 5% or more of body weight over the preceding 12 months. As this variable was not collected in the 2023 NSOK, we substituted a proxy measure of unintentional weight change of 5 kg or more within the prior 6 months. This modification should be considered when comparing our results with studies using the original definition.

### 4.6 Future directions

This study demonstrated the feasibility of explainable AI for frailty risk prediction using nationally representative survey data. However, several steps are required to advance toward robust and implementable systems. First, external validation across multiple sites and time periods is essential to assess generalizability. Such research should incorporate sampling weights to ensure population representativeness and apply recalibration strategies when transportability gaps are identified. Second, because frailty is a dynamic process, future research should adopt survival or longitudinal designs (e.g., Cox proportional hazards models, landmarking, and joint models) to evaluate predictive stability over time. Third, decision thresholds should move beyond conventional metrics and be optimized through cost–benefit analyses, with threshold selection guided by approaches such as the Youden index (13, 14), thereby linking predictions to real-world intervention trade-offs. Fourth, systematic assessment of subgroup fairness (considering factors such as sex, age, and socioeconomic status) and bias mitigation strategies is needed. Additionally, user acceptance testing should be conducted to determine whether explainability outputs genuinely improve trust and clinical decision-making (51). Finally, integration of wearable devices, electronic health records, and personal health records may enable continuous and personalized frailty risk monitoring, supporting adaptive interventions in public health and primary care (5, 6, 55).

## 5 Conclusion

This study developed and internally validated an explainable artificial intelligence (XAI) framework for frailty risk prediction using nationally representative survey data. By integrating SHAP-based feature interpretation with ensemble algorithm comparison, the framework demonstrated that predictive performance and interpretability can be achieved concurrently in a population-based context.

The findings suggest that frailty risk is shaped by interrelated medical, functional, psychological, social, and digital factors rather than by chronological aging alone. These results underscore the importance of incorporating multidomain information into future frailty screening and prevention strategies. The lightweight web application developed in this study serves as a proof of concept for translating explainable AI models into accessible tools for use in community and primary care settings.

Although limited by its cross-sectional design, relatively small frail subgroup, and lack of external validation, this study presents a reproducible and transparent framework for applying explainable machine learning to public-health data. Future research should build upon this research by conducting external and longitudinal validation, assessing recalibration needs, and optimizing thresholds to enhance model generalizability and clinical applicability.

Overall, this study contributes to the growing body of evidence that explainable AI is a feasible and interpretable approach for population-level frailty research and prevention, aligning with the goals of the WHO and United Nations Decade of Healthy Ageing 2021–2030 initiatives (52, 53).

## Data Availability

Publicly available datasets were analyzed in this study. This data can be found here: the datasets are available in the Korea Institute for Health and Social Affairs (KIHASA) data repository. Specifically, we used the 2023 Korean National Survey of Older Persons, conducted by the Ministry of Health and Welfare. The data can be accessed at https://data.kihasa.re.kr. No accession number is applicable, as access requires registration and approval through the KIHASA system.
