# Supplementary material for: Development of machine learning models with explainable AI for frailty risk prediction and their web-based application in community public health
Source: Front Public Health. 2025 Nov 6;13:1698062. doi: 10.3389/fpubh.2025.1698062 (PMC12629939; doi:10.3389/fpubh.2025.1698062)
Supplement: Supplementary file 2 [file Data_Sheet_1.PDF]

Table S2. Hyperparameter Search Space for 10 Machine Learning Models

Model	Hyperparameters	Search Range / Values	Status
**Logistic Regression**	C	[0.1, 1.0, 10.0]	Search
	class_weight	[None, "balanced"]	Search
	solver	"liblinear"	Fixed
**SVM (RBF)**	C	[0.5, 1.0, 2.0]	Search
	gamma	"scale"	Fixed
**KNN**	n_neighbors	[5, 15, 31]	Search
	weights	["uniform", "distance"]	Search
**MLP**	hidden_layer_sizes	[(64,), (128,)]	Search
	alpha	[1e-4, 1e-3]	Search
	max_iter	300	Fixed
**Random Forest**	n_estimators	[300]	Fixed
	max_depth	[None, 10]	Search
	max_features	["sqrt"]	Fixed
**Gradient Boosting**	n_estimators	[300]	Fixed
	learning_rate	[0.05, 0.1]	Search
	max_depth	[2, 3]	Search
**HistGradientBoosting**	max_depth	[None, 10]	Search
	learning_rate	[0.05, 0.1]	Search
	max_iter	[300]	Fixed
**XGBoost**	max_depth	[3, 5]	Search
	learning_rate	[0.05, 0.1]	Search
	subsample	[0.8]	Fixed
	colsample_bytree	[0.8]	Fixed
**LightGBM**	num_leaves	[31, 63]	Search
	learning_rate	[0.05, 0.1]	Search
	subsample	[0.8]	Fixed
	colsample_bytree	[0.8]	Fixed
**CatBoost**	depth	[6]	Fixed
	learning_rate	[0.05, 0.1]	Search
	iterations	[300]	Fixed
