# Supplementary material for: Development of machine learning models with explainable AI for frailty risk prediction and their web-based application in community public health
Source: Front Public Health. 2025 Nov 6;13:1698062. doi: 10.3389/fpubh.2025.1698062 (PMC12629939; doi:10.3389/fpubh.2025.1698062)
Supplement: Supplementary file 3 [file Data_Sheet_2.PDF]

Supplementary Table 3. Missing Codes and Distribution of Original Variables in the 2023 Korean National Survey of Older Koreans

Label	Original_Code	Missing_Codes	Count_as_Missing	Rate(%)
**Marital_Status**	RES_MAR	9	0	0
**Education_Level**	RES_EDU1	99	0	0
**Self_Rated_Health**	B1	9	127	1.26
**Sleep_Quality**	B2	9	127	1.26
**Height**	B7_1	999, 9999	0	0
**Weight**	B7_2	998	1	0.01
**Weight_Change_6mo**	B13_11	9	0	0
**Suicidal_Ideation**	B9	9	127	1.26
**Unmet_Medical_Needs_Hospital**	B16_1	9	127	1.26
**Unmet_Medical_Needs_Dental**	B16_2	9	127	1.26
**Hypertension**	B3_2L_1	8, 9	0	0
**Stroke**	B3_2L_2	8, 9	0	0
**Hyperlipidemia**	B3_2L_3	8, 9	0	0
**Angina**	B3_2L_4	8, 9	0	0
**Myocardial_Infarction**	B3_2L_5	8, 9	0	0
**Other_Heart_Disease**	B3_2L_6	8, 9	0	0
**Diabetes**	B3_2L_7	8, 9	0	0
**Thyroid_Disease**	B3_2L_8	8, 9	0	0
**Arthritis**	B3_2L_9	8, 9	0	0
**Osteoporosis**	B3_2L_10	8, 9	0	0
**Back_Pain**	B3_2L_11	8, 9	0	0
**Fracture_Sequelae**	B3_2L_12	8, 9	0	0
**Chronic_Respiratory_Disease**	B3_2L_13	8, 9	0	0
**Asthma**	B3_2L_14	8, 9	0	0
**Tuberculosis**	B3_2L_15	8, 9	0	0
**Depression_Diagnosed**	B3_2L_16	8, 9	0	0
**Dementia**	B3_2L_17	8, 9	0	0
**Parkinson**	B3_2L_18	8, 9	0	0
**Insomnia**	B3_2L_19	8, 9	0	0
**Cataract**	B3_2L_20	8, 9	0	0
**Glaucoma**	B3_2L_21	8, 9	0	0
**Chronic_Otitis_Media**	B3_2L_22	8, 9	0	0
**Presbycusis**	B3_2L_23	8, 9	0	0

**Skin_Disease**	B3_2L_24	8, 9	0	0
**Cancer**	B3_2L_25	8, 9	0	0
**Peptic_Ulcer**	B3_2L_26	8, 9	0	0
**Hepatitis**	B3_2L_27	8, 9	0	0
**Cirrhosis**	B3_2L_28	8, 9	0	0
**Chronic_Kidney_Disease**	B3_2L_29	8, 9	0	0
**Prostatic_Hyperplasia**	B3_2L_30	8, 9	0	0
**Incontinence**	B3_2L_31	8, 9	0	0
**Anemia**	B3_2L_32	8, 9	0	0
**Other_Chronic_Disease**	B3_2L_33	8, 9	0	0
**Depression_Life_Satisfaction**	B8_1	9	127	1.26
**Depression_Loss_Motivation**	B8_2	9	127	1.26
**Depression_Vanity**	B8_3	9	127	1.26
**Depression_Boredom**	B8_4	9	127	1.26
**Depression_Refreshing**	B8_5	9	127	1.26
**Depression_Anxiety**	B8_6	9	127	1.26
**Depression_Pleasure**	B8_7	9	127	1.26
**Depression_Hopelessness**	B8_8	9	127	1.26
**Depression_Avoid_Going_Out**	B8_9	9	127	1.26
**Depression_Poor_Memory**	B8_10	9	127	1.26
**Depression_Life_Joy**	B8_11	9	127	1.26
**Depression_Worthlessness**	B8_12	9	127	1.26
**Depression_Energetic**	B8_13	9	127	1.26
**Depression_Hopeless**	B8_14	9	127	1.26
**Depression_Self_Degradation**	B8_15	9	127	1.26
**ADL_Dressing**	C7_1	8, 9	0	0
**ADL_Wash**	C7_2	8, 9	0	0
**ADL_Bathing**	C7_3	8, 9	0	0
**ADL_Eating**	C7_4	8, 9	0	0
**ADL_Moving**	C7_5	8, 9	0	0
**ADL_Toileting**	C7_6	8, 9	0	0
**ADL_Continence**	C7_7	8, 9	0	0
**IADL_Grooming**	C8_1_1	8, 9	0	0
**IADL_Housework**	C8_1_2	8, 9	0	0
**IADL_Preparing_Meal**	C8_1_3	8, 9	0	0
**IADL_Laundry**	C8_1_4	8, 9	0	0

**IADL_Taking_Medication**	C8_1_5	8, 9	0	0
**IADL_Finance_Management**	C8_1_6	8, 9	0	0
**IADL_Short_Trip**	C8_1_7	8, 9	0	0
**IADL_Shopping**	C8_2_8	8, 9	0	0
**IADL_Telephone**	C8_2_9	8, 9	0	0
**IADL_Transportation**	C8_2_10	8, 9	0	0
**Num_Medications**	B4	8, 9	49	0.49
**Hospital_Use**	B5	8, 9	0	0
**Current_Smoking**	B10	8, 9	0	0
**Alcohol_Frequency**	B11	8, 9	0	0
**Physical_Activity**	B12	8, 9	0	0
**Dietary_Control**	B13_1	8, 9	0	0
**Meal_Less_Than_Twice**	B13_2	8, 9	0	0
**Frequent_Drinking**	B13_6	8, 9	0	0
**Eating_Alone**	B13_9	8, 9	0	0
**Polypharmacy**	B13_10	8, 9	0	0
**Health_Checkup**	B17_1	8, 9	0	0
**Fall_Experience**	C6	8, 9	0	0
**Disability_Diagnosis**	C11	8, 9	0	0
**Internet_Access_At_Home**	D10	8, 9	0	0
**Digital_Adaptation_Difficulty**	D13	8, 9	127	1.26
**Owns_Smartphone**	D11_1_1	8, 9	0	0
**Owns_Computer**	D11_1_2	8, 9	0	0
**Owns_Smartwatch**	D11_1_3	8, 9	0	0
**Uses_Smartphone**	D11_2_1	8, 9	0	0
**Uses_Computer**	D11_2_2	8, 9	0	0
**Uses_Smartwatch**	D11_2_3	8, 9	0	0
**Economic_Activity**	E1	9	0	0
**Living_With_Children**	G1_1	8, 9	0	0
**Household_Type**	H1	8, 9	0	0
**Housing_Satisfaction**	H2	8, 9	127	1.26
**Transportation**	H9	8, 9	0	0
**Driving**	H11	8, 9	0	0
**Voted_in_Elections_Last_3_Years**	H24	8, 9	127	1.26
**Basic_Livelihood_Benefit**	J2	99998, 99999	0	0
**Household_Income**	J3b_3_13	99998, 99999	0	0

**Household_Expenditure**	J4	99998, 99999	0	0
**Household_Housing_Expenditure**	J4_1	99998, 99999	0	0
**Median_Income_Ratio**	TJ3K13H_3_2	99998, 99999	0	0
**Cognition_Total_Score**	QIK2	force_numeric	0	0
**Fatigue**	C3	8, 9	127	1.26
**Climb_Stairs_Difficulty**	C4	8, 9	0	0
**Walk_300m_Difficulty**	C5	8, 9	0	0
