# Supplementary figures and images for: Development of machine learning models with explainable AI for frailty risk prediction and their web-based application in community public health
Source: Front Public Health. 2025 Nov 6;13:1698062. doi: 10.3389/fpubh.2025.1698062 (PMC12629939; doi:10.3389/fpubh.2025.1698062)

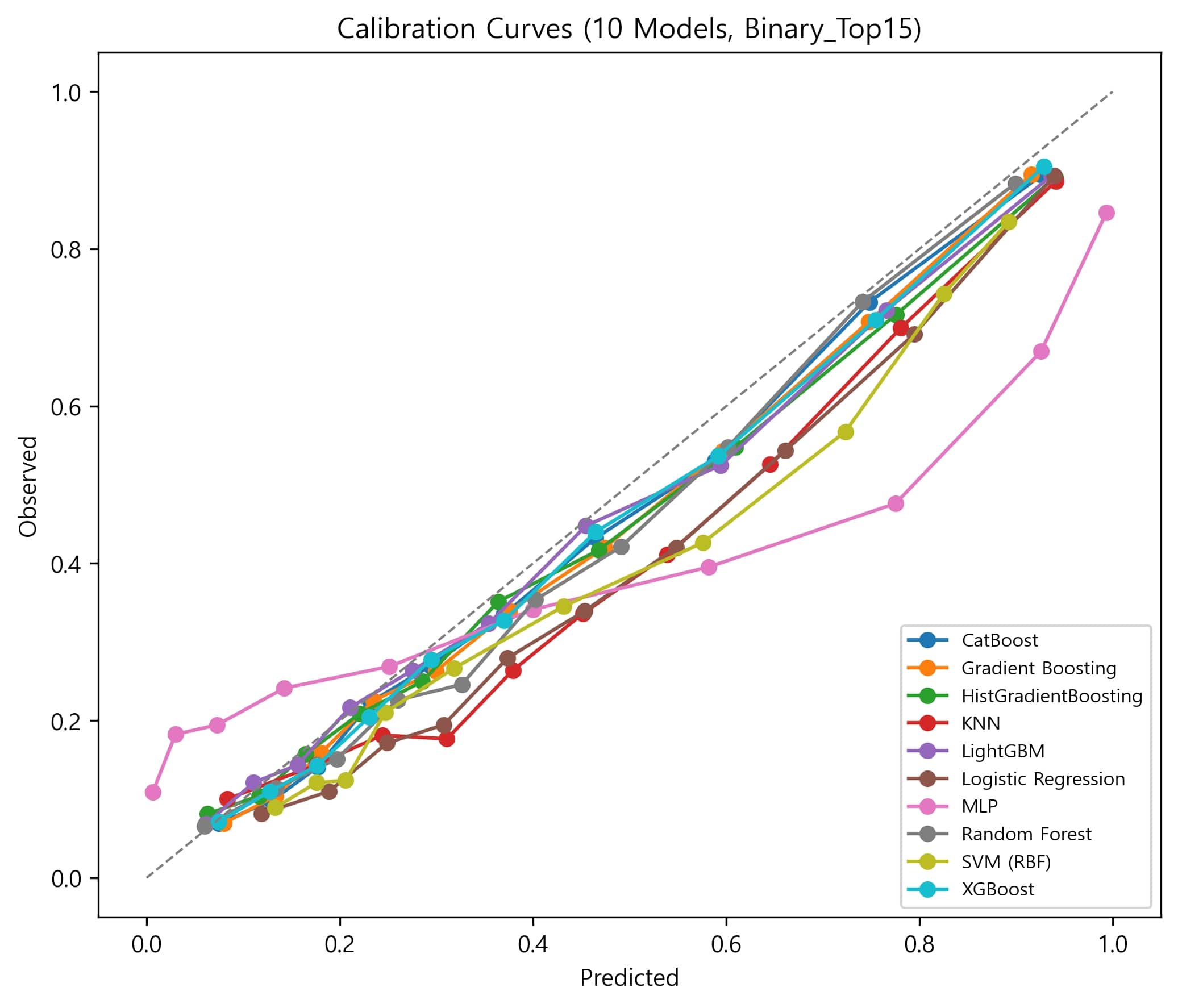

Supplement: Supplementary file 4 [file Image_1.JPEG]

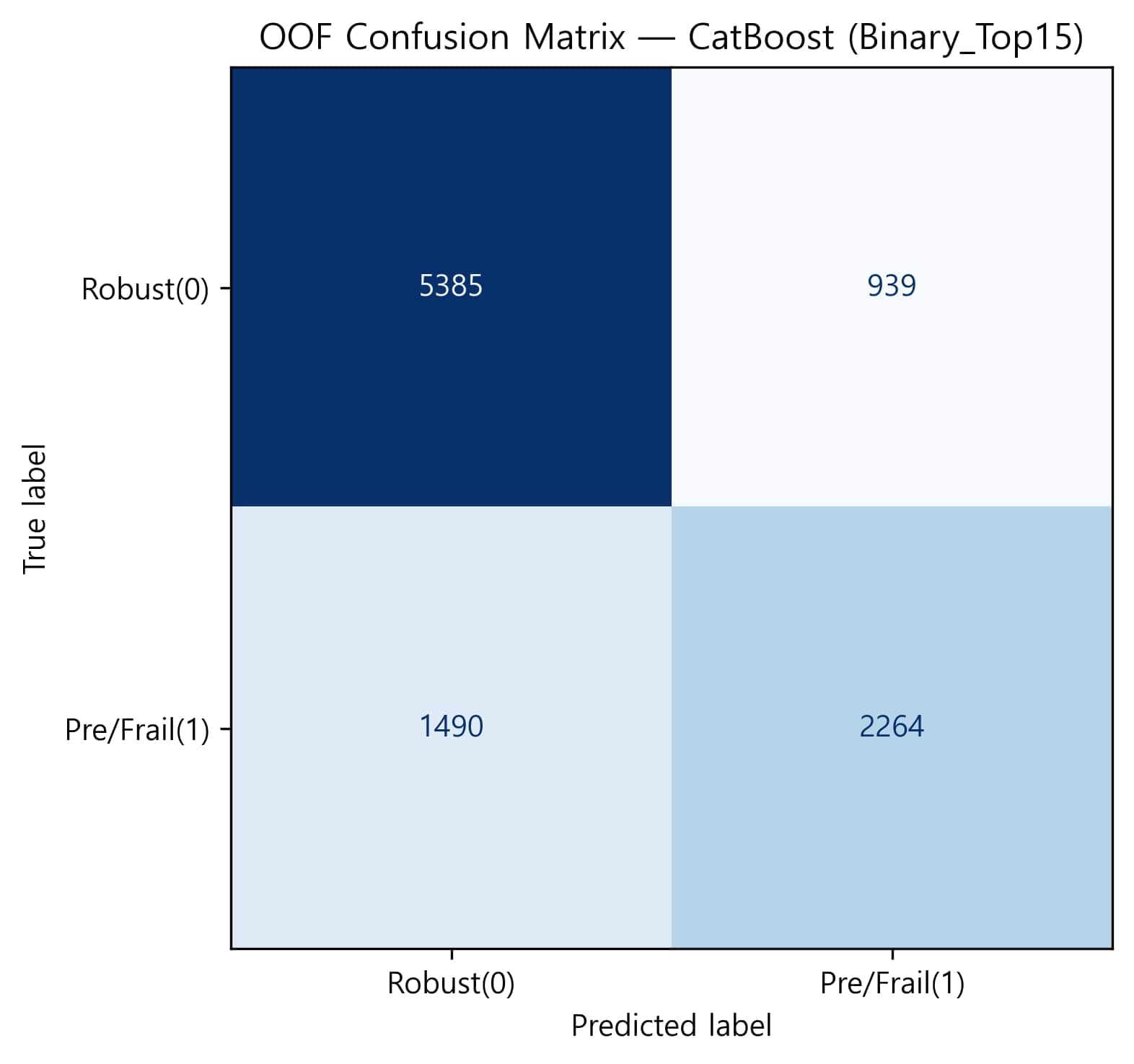

Supplement: Supplementary file 5 [file Image_2.JPEG]

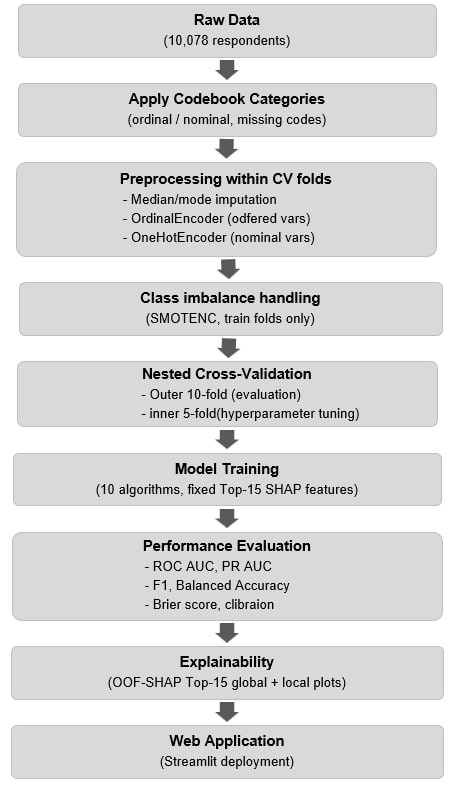

Supplement: Supplementary file 6 [file Image_3.JPEG]
